# Supplementary material for: BART Streams: Real‐Time Reconstruction Using a Modular Framework for Pipeline Processing
Source: Magn Reson Med. 2026 Jun 4;96(4):1534–44. doi: 10.1002/mrm.70455 (PMC13419017; doi:10.1002/mrm.70455)
Supplement: Supplementary file 1 — Data S1: Real‐time MRI reconstruction pipeline graph. Figure S1: State‐of‐the‐art reconstruction pipeline for real‐time MRI. Different sections of the reconstruction pipeline are highlighted, and characteristic parts are also shown in a zoomed‐in, abbreviated version next to the full graph. Round nodes represent BART invocations while edges between tool nodes represent pipes. The invocation of the real‐time reconstruction script corresponding to this graph is shown at the bottom, along with an explanation of command line options. The figure is based on an auto‐generated graph, which was created by tracing every BART tool invocation and translating this trace into a graph description language, and was rendered with dot. [file MRM-96-1534-s002.pdf]

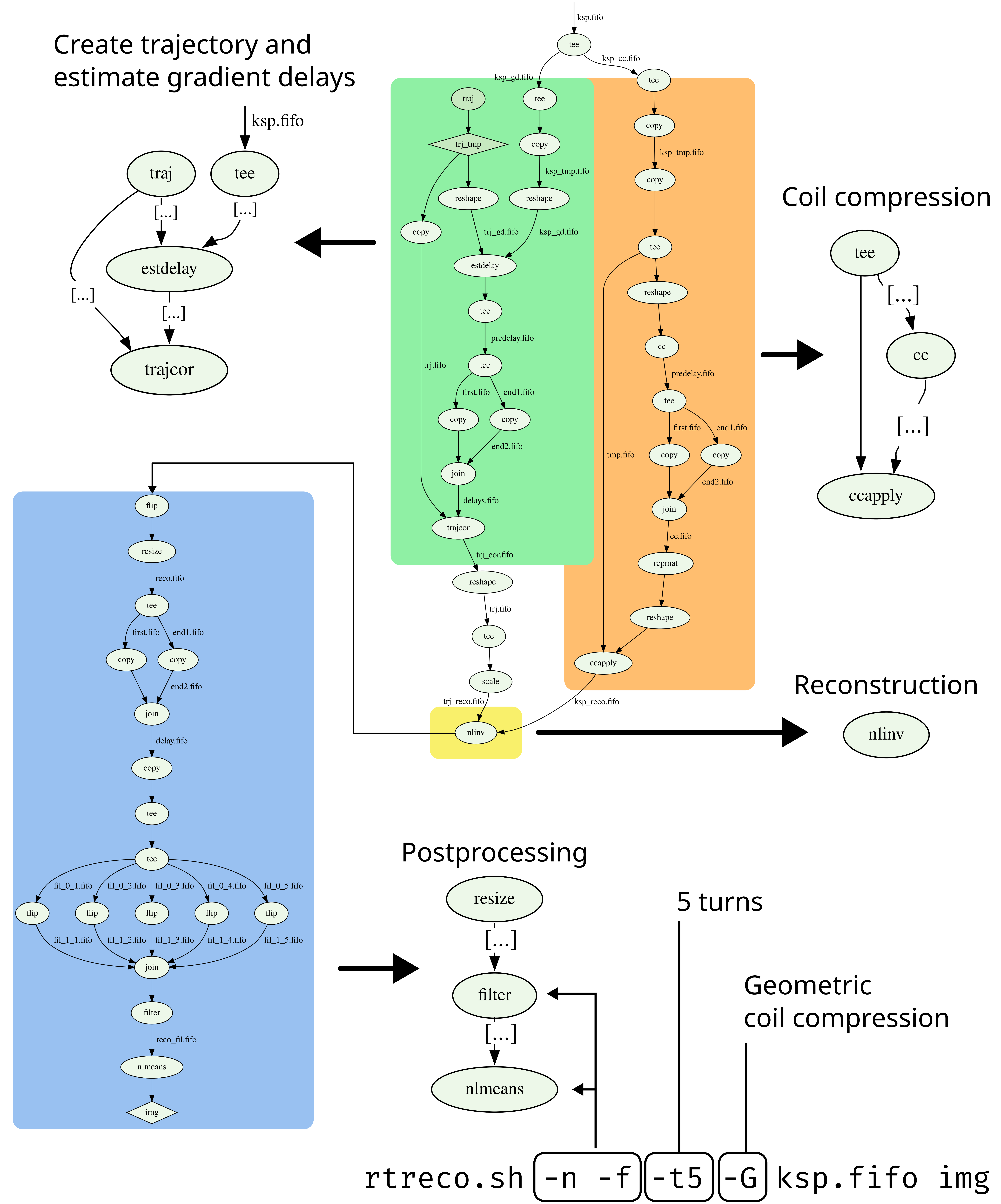

Create trajectory and
estimate gradient delays
ksp.fifo
traj
tee
[...]
estdelay
trajcor
Coil compression
cc
ccapply
Reconstruction
nlinv
Postprocessing
resize
filter
nlmeans
5 turns
Geometric
coil compression
ksp_gd.fifo
ksp_cc.fifo
trj_tmp
copy
reshape
ksp_tmp.fifo
trj_gd.fifo
predelay.fifo
trj.fifo
first.fifo
end1.fifo
end2.fifo
join
delays.fifo
trj_cor.fifo
scale
trj_reco.fifo
tmp.fifo
cc.fifo
repmat
ksp_reco.fifo
flip
reco.fifo
delay.fifo
fil_0_1.fifo
fil_0_2.fifo
fil_0_3.fifo
fil_0_4.fifo
fil_0_5.fifo
fil_1_1.fifo
fil_1_2.fifo
fil_1_3.fifo
fil_1_4.fifo
fil_1_5.fifo
reco_fil.fifo
img
rtreco.sh -n -f -t5 -G ksp.fifo img
