# Supplementary material for: BART Streams: Real‐Time Reconstruction Using a Modular Framework for Pipeline Processing
Source: Magn Reson Med. 2026 Jun 4;96(4):1534–44. doi: 10.1002/mrm.70455 (PMC13419017; doi:10.1002/mrm.70455)
Supplement: Supplementary file 2 — Data S2: End‐to‐end latency measurement. Figure S2: Part A: Schematic overview of the end‐to‐end latency measurement experiment setup. A water‐filled test‐tube (blue) inside the MRI scanner is pulled up from the control room using a long string (yellow). Images from the real‐time sequence are shown on the MRI console screen. A smartphone camera records movement on the MRI screen and movement of a paper tag (gray) attached to the string. A single frame from the resulting video is shown on the top right. Part B illustrates how the resulting video is processed: The video is segmented, and the obtained object velocity curves are cross‐correlated to obtain the delay between both movements. [file MRM-96-1534-s003.pdf]

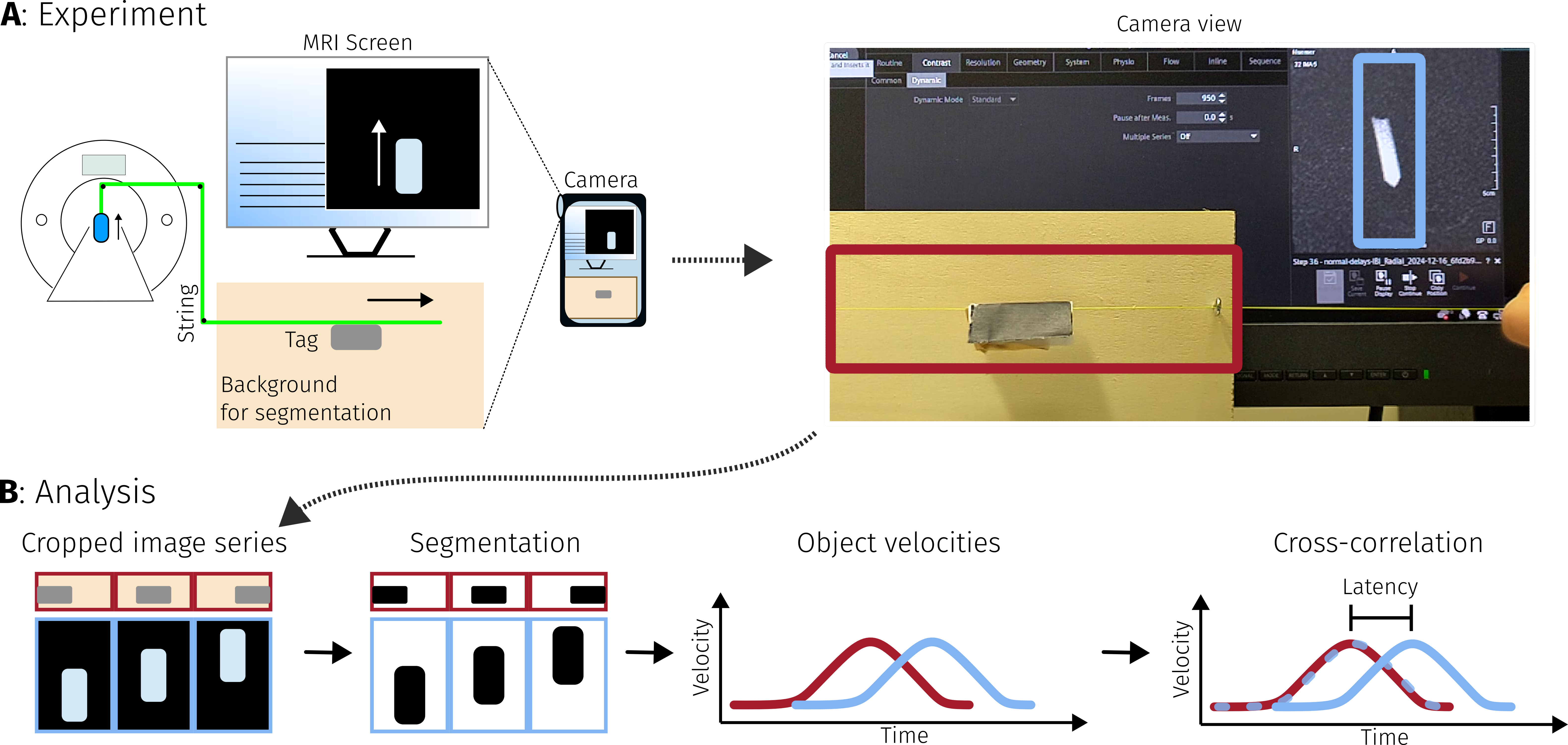

A: Experiment
MRI Screen
Camera
Camera view
String
Tag
Background
for segmentation
B: Analysis
Cropped image series
Segmentation
Object velocities
Cross-correlation
Latency
Velocity
Time
Velocity
Time
